# Unveiling pterion variability: a meta-analytic approach to enhance neurosurgical precision

**DOI:** 10.1007/s00276-025-03571-6

**Published:** 2025-01-28

**Authors:** George Triantafyllou, Nektaria Karangeli, Fabrice Duparc, Maria Piagkou, Renato Galzio, George Tsakotos, George Botis, Theodosis Kalamatianos, Sabino Luzzi

**Affiliations:** 1 Department of Anatomy, School of Medicine, Faculty of Health Sciences, National and Kapodistrian University of Athens, 75 Mikras Asias str, Goudi, Athens, 11527 Greece; 2 Department of Anatomy, Faculty of Medicine-Pharmacy, University of Rouen-Normandy, Rouen-Normandy, France; 3 Department of Clinical-Surgical, Diagnostic and Pediatric Sciences, University of Pavia, Pavia, Italy; 4 Biomedical Engineering Laboratory, School of Electrical and Computer Engineering, National Technical University of Athens, Athens, Greece; 5 Department of Neurosurgery, Evangelismos Hospital, School of Medicine, Faculty of Health Sciences, National and Kapodistrian University of Athens, Athens, Greece

**Keywords:** Pterion, Pterional variability, Morphology, Neurosurgery, Pterional approach, Evidence-based anatomy

## Abstract

**Purpose:**

This meta-analytical systematic review aims at investigating the variability of the pterion, focusing on its morphological types and precise distances from various bony landmarks. Additionally, the neurosurgical significance of this critical cranial landmark is examined in depth.

**Methods:**

The systematic review was conducted following PRISMA 2020 and Evidence-based Anatomy Workgroup guidelines for anatomical studies. The risk of bias was assessed using the Anatomical Quality Assurance Tool (AQUA). The meta-analysis was performed using R programming software and RStudio, employing the “meta” and “metafor” packages.

**Results:**

A total of 79 studies were included, encompassing 18,694 skull sides. The sphenoparietal type was identified as the most prevalent pterion variant, with a pooled prevalence of 78.54%. The epipteric type followed at 8.41%, while the frontotemporal (5.74%) and stellate (4.26%) types were the rarest. Significant differences in the prevalence of the epipteric, frontotemporal, and stellate types were observed across different nationalities. However, sex, side, and study type did not significantly influence pterion morphological variation. The study also extracted and calculated the distances between the pterion and key anatomical landmarks, including the midpoint of the zygomatic arch, the frontozygomatic suture, the mastoid process, and the external acoustic meatus.

**Conclusion:**

This study offers critical anatomical insights by accurately mapping the pterion’s location relative to essential cranial landmarks. These findings are vital for neurosurgical planning, particularly for procedures involving the anterior and middle cranial fossae. The detailed anatomical data provided can enhance the precision and safety of neurosurgical interventions, ultimately improving patient outcomes.

**Supplementary Information:**

The online version contains supplementary material available at 10.1007/s00276-025-03571-6.

## Introduction

The pterion (PT) is considered one of the most important skull landmarks. It is located in the temporal fossa, forming an irregular H-shaped small circular area. The significance of this craniometric point lies in its attribute as the meeting point of the frontal bone, the sphenoidal (anteroinferior) angle of the parietal bone, the greater sphenoidal wing, and the temporal bone’s squamous part. Hence, this junction is where the coronal, sphenoparietal, sphenofrontal, sphenosquamous, and squamous sutures converge [79]. In neonatal skulls, this articulation coincides with the anterolateral (sphenoid) fontanelle, which is estimated to vanish 3–6 months after birth [55, 79].

PT is frequently referenced as a critical region for various brain structures. Among the most notable ones are the anterior branch of the middle meningeal artery, the Broca’s area (dedicated to motor aspects of speech), the insula, the lateral sulcus (Sylvian cerebral sulcus), the anterior Sylvian point, and the anterior cisterns of the encephalon base. These anatomical relationships highlight the PT’s primary significance in the neurosurgical field [19, 38–40, 57]. The pterional approach introduced by Yasargil et al. [91] has been established as an extensively utilized technique with multiple targets, both vascular (e.g., internal carotid artery, middle cerebral artery) and tumorous (e.g., orbital, insula, basal ganglia, and uncus).

**Fig. 1:**
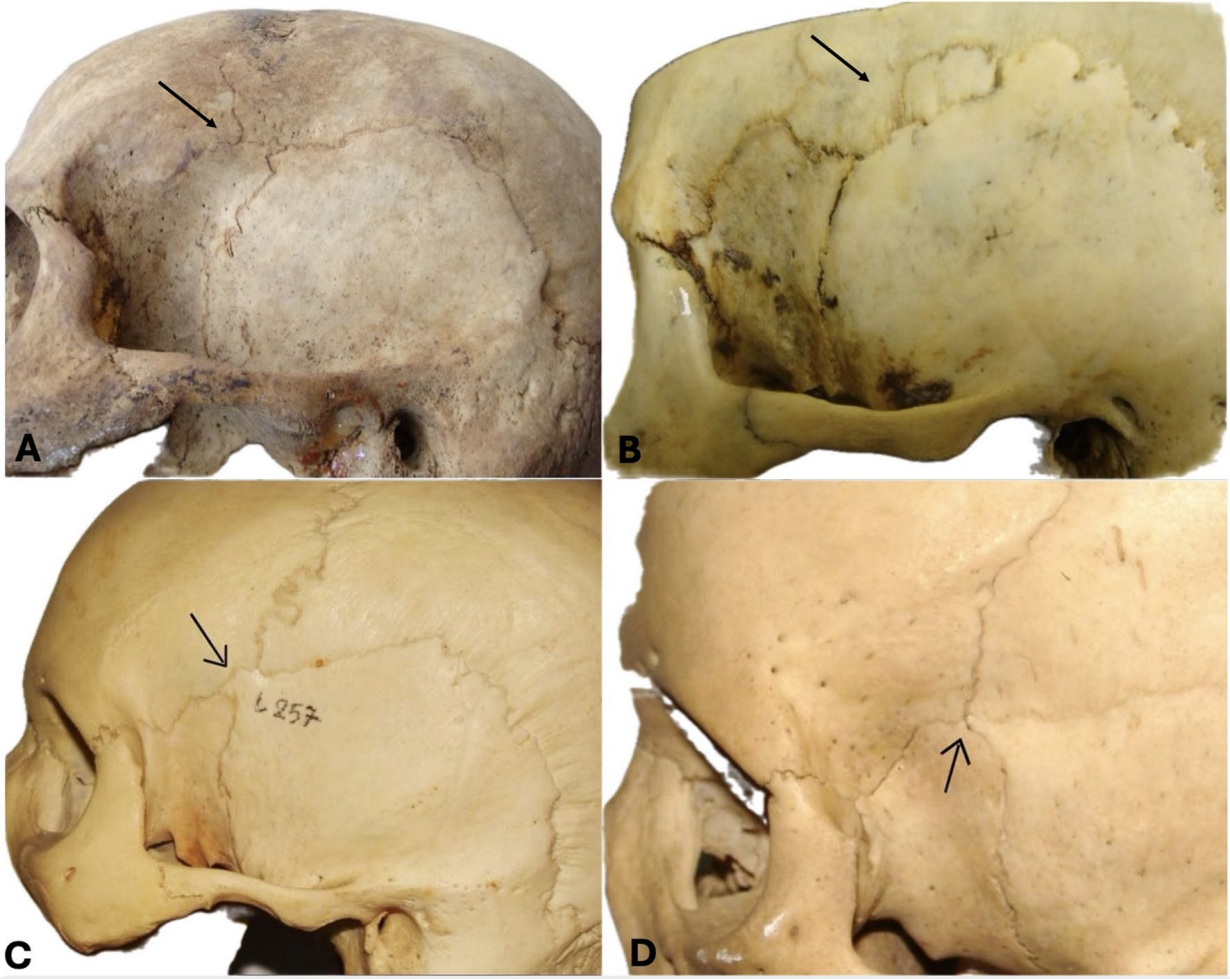
Murphy [50] proposed four morphological types of pterion. (**A**) The sphenoparietal type; (**B**) The epipteric type; (**C**) The frontotemporal type; (**D**) The stellate type

The PT morphological and morphometric characteristics exhibit significant variability. Murphy [50] identified four distinct morphological variants: sphenoparietal, frontotemporal, stellate, and epipteric (Fig. 1). The epipteric variant demonstrates further variability regarding the number and configuration of the epipteric bones (EBs) [55]. Additionally, the PT distance from other significant anatomical landmarks shows considerable differences across individuals, and factors such as sex and ethnicity are pivotal [55, 82].

While several previous anatomical studies have examined PT morphology and localization, the current literature lacks an extensive review that exploits the existing data. By systematically analyzing the different morphological types and their morphometric distances from key cranial landmarks, this research provides a more comprehensive understanding of the pterion’s anatomical variations, which is essential for precise surgical planning and for reducing the risk of complications in neurosurgical interventions.

## Materials and methods

The Evidence-based Anatomy Workgroup [26] and the PRISMA 2020 [59] guidelines were followed for the current systematic review with meta-analysis. Moreover, to detect possible publication bias, the Anatomical Quality Assurance Tool (AQUA) was used [27]. Five domains with questions and possible answers of “Yes, No, or Unclear” provide the potential risk of bias as “Low, High, or Unclear.”

Four independent reviewers performed the literature search and data extraction. Results were compared, and the other authors settled potential differences. The terms “pterion,” “morphology,” “variation,” “variability,” “anatomical study,” “imaging study,” and “neurosurgical study” were used in different combinations in the online databases PubMed, Google Scholar, Scopus, and *Web of Science*, until July 2024. Studies reporting on the PT morphology and morphometry were selected as eligible. Regarding PT morphometry, the following distances were identified from the included studies: distance from the midpoint of the zygomatic arch (MPZ), from the frontozygomatic suture (FZMS), from the mastoid process (MP), and from the external acoustic meatus (EAM). Exclusion criteria were case reports, animal studies, conference abstracts and letters to the editor, and studies with irrelevant, insufficient, or incomplete data; however, no language or date restrictions were imposed. Other resources were investigated to identify more eligible articles. A hands-on search of the grey literature and the significant anatomical journals (*Annals of Anatomy*,* Clinical Anatomy*,* Journal of Anatomy*,* Anatomical Record*,* Surgical and Radiological Anatomy*,* Folia Morphology*,* Anatomical Science International*,* European Journal of Anatomy*,* Anatomy and Cell Biology*) was also performed. Lastly, the references of all included studies for additional articles were investigated. The data were extracted using Microsoft Excel sheets before statistical analysis.

Statistical analysis was conducted using the open-source R programming language and the RStudio software version 4.3.2 using the “meta” and “metafor” packages. The pooled prevalence was calculated using the inverse variance and random effects models. The proportions meta-analysis (prevalence meta-analysis) was conducted using the Freeman-Tukey double arcsine transformation, the DerSimonian-Laird estimator for the between-study variance tau^2^, and the Jackson method for confidence interval of tau^2^ and tau. The means (mean distances) meta-analysis was conducted using the untransformed (raw) means, the restricted maximum-likelihood estimator for tau^2^, and the Q-Profile method for confidence interval of tau^2^ and tau. A p-value less than 0.05 was considered statistically significant. Cochran’s Q statistic was used to evaluate the presence of heterogeneity across studies, and the Higgins I^2^ statistic was used to quantify heterogeneity. Cochran’s Q p-value < 0.10 was considered significant. Higgins I^2^ values between 0 and 40% were regarded as not necessary, 30–60% as moderate heterogeneity, 50–90% characterized as substantial heterogeneity, and 75–100% may represent considerable heterogeneity. To evaluate the presence of a small-study effect (the phenomenon that smaller studies may show different effects than large ones), the DOI plot with the LFK index was generated [21].

## Results

**Fig. 2:**
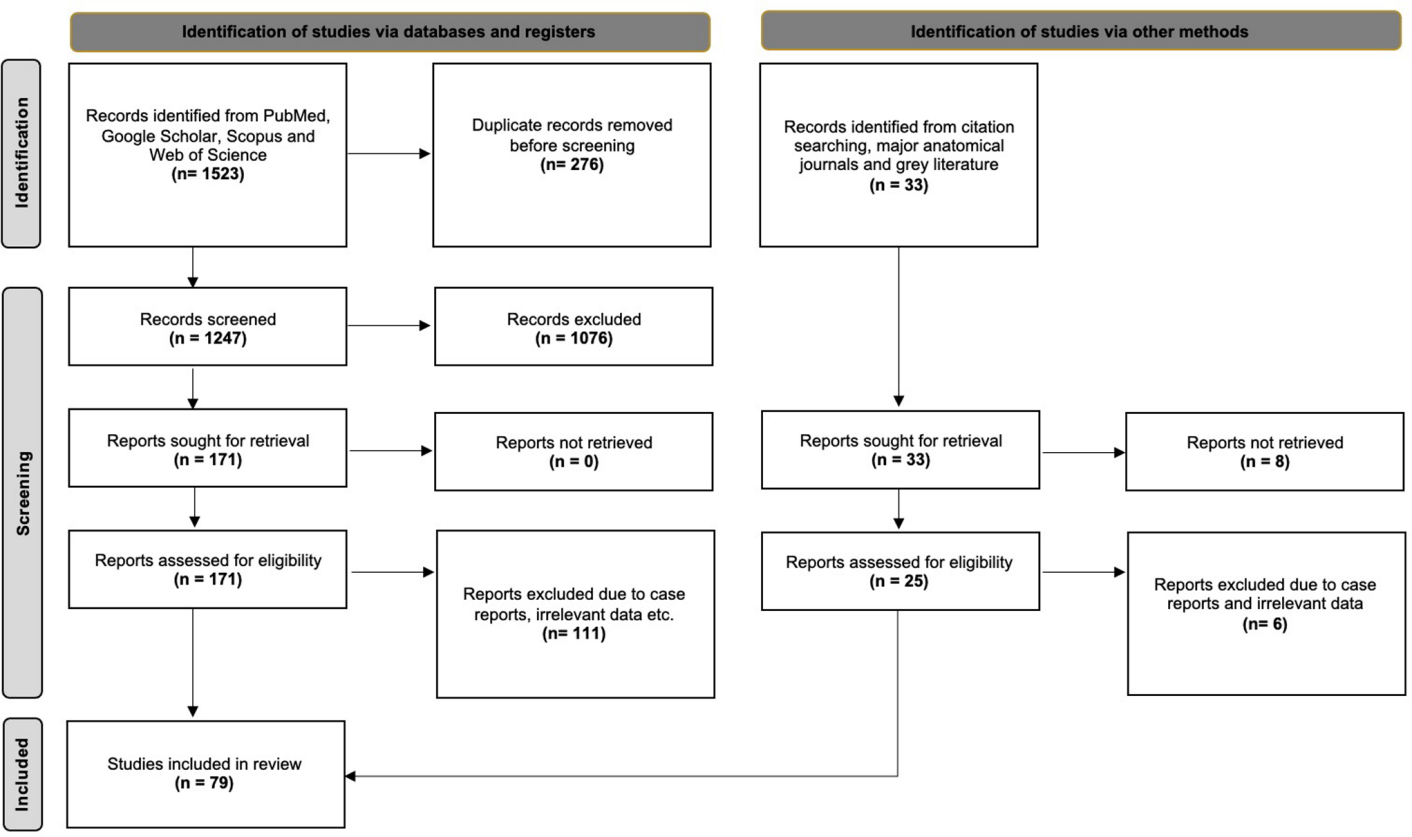
PRISMA 2020 flow chart of search analysis [59]

The initial database results produced 1,523 articles exported to Mendeley version 2.10.9 (*Elsevier*,* London*). Firstly, a duplicate check was performed, and the titles and abstracts were assessed for eligibility. After excluding irrelevant papers, 171 studies underwent full-text retrieval and screening. Finally, 60 studies were considered eligible for our initial meta-analysis questions. Furthermore, 19 studies were identified from references, grey literature, and significant anatomical journals through a hands-on search. Hence, 79 studies were included in the current systematic review with meta-analysis. Following the PRISMA 2020 guidelines [59], Fig. 2 presents the flow diagram of the selection process.

Seventy-nine studies were included, with a sample of 18.694 skull sides. Only two studies were imaging, while the rest (77 studies) were dried skull studies. Forty-eight (48) studies had a sample of more than 100 skull sides, while 31 studies had a sample of less than 100 skull sides. The mean sample per article was 236.65 skull sides. Sixty-four studies were based on Asian populations, 10 on African populations, 2 studies on Australian and European, and only one study on an American population (Table 1). Most studies evaluated PT morphology according to Murphy’s classification system [50], while the morphometric details (distances from significant landmarks) varied among the studies in the literature.

**Table 1:** Summary of the included studies. The risk of bias was assessed with the Anatomical Quality Assessment Tool (AQUA) Tool^13^. NR- not reported

Study	Year	Population	Type of Study	No. of skulls	Risk of bias
Murphy [50]	1956	Australia	Cadaveric	368	Low
Agarwal et al. [2]	1980	Asia	Cadaveric	450	High
Saxena et al. [72]	1988	Asia	Cadaveric	72	High
Saxena et al. [72]	1988	Africa	Cadaveric	40	High
Matsumura et al. [45]	1991	Asia	Cadaveric	614	High
Asala and Mbajiorgu [10]	1996	Africa	Cadaveric	212	High
Lee et al. [36]	2001	Asia	Cadaveric	149	High
Ersoy et al. [18]	2003	Asia	Cadaveric	330	High
Saxena et al. [71]	2003	Asia	Cadaveric	203	High
Oguz et al. [57]	2004	Asia	Cadaveric	26	High
Ilknur et al. [29]	2009	Asia	Cadaveric	44	High
Hussain Saheb et al. [28]	2010	Asia	Cadaveric	125	High
Mwachaka et al. [51]	2010	Africa	Cadaveric	90	Low
Zalawadia et al. [92]	2010	Asia	Cadaveric	42	High
Apinhasmit et al. [9]	2011	Asia	Cadaveric	268	Low
Bhargavi et al. [11]	2011	Asia	Cadaveric	70	High
Natekar et al. [54]	2011	Asia	Cadaveric	150	High
Ma et al. [41]	2012	Asia	Cadaveric	76	Low
Ma et al. [41]	2012	Australia	Imaging	50	Low
Praba and Venkatramaniah [61]	2012	Asia	Cadaveric	50	High
Adejuwon et al. [1]	2013	Africa	Cadaveric	37	High
Kumar et al. [35]	2013	Asia	Cadaveric	40	High
Sudha et al. [80]	2013	Asia	Cadaveric	150	High
Ukoha et al. [84]	2013	Africa	Cadaveric	56	High
Aksu et al. [5]	2014	Asia	Cadaveric	128	High
Eboh and Obaroefe [17]	2014	Africa	Cadaveric	50	High
Mahajan [42]	2014	Asia	Cadaveric	50	High
Nair et al. [53]	2014	Asia	Cadaveric	500	Low
Anjana et al. [8]	2015	Asia	Cadaveric	32	High
Havaldar et al. [25]	2015	Asia	Cadaveric	250	High
Satpute and Wahane [70]	2015	Asia	Cadaveric	85	High
Sindel et al. [77]	2016	Asia	Cadaveric	150	High
Walulkar et al. [88]	2016	Asia	Cadaveric	350	High
Warille and Mandloi [89]	2016	Asia	Cadaveric	71	High
Dutt et al. [16]	2017	Asia	Cadaveric	78	High
Rao et al. [66]	2017	Asia	Cadaveric	70	High
Gindha et al. [23]	2017	Asia	Cadaveric	65	High
Khaleel et al. [33]	2017	Asia	Cadaveric	250	High
Manjunath and Channabasanagouda [44]	2017	Asia	Cadaveric	282	High
Nayak et al. [56]	2017	Asia	Cadaveric	50	High
Vasudha et al. [85]	2017	Asia	Cadaveric	150	High
Wadekar et al. [87]	2017	Asia	Cadaveric	55	High
Alam [6]	2018	Asia	Cadaveric	50	High
Modasiya and Kanani [48]	2018	Asia	Cadaveric	110	High
Chandran and Mohanra [12]	2019	Asia	Cadaveric	50	High
Cimen et al. [13]	2019	Asia	Cadaveric	75	High
Francis et al. [19]	2019	Asia	Cadaveric	50	High
Kamath and Hande [31]	2019	Asia	Cadaveric	50	Low
Mehra and Kumar [46]	2019	Asia	Cadaveric	40	High
Mehta et al. [47]	2019	Asia	Cadaveric	31	High
Natsis et al. [55]	2019	Europe	Cadaveric	90	Low
Patil and Kumar [60]	2019	Asia	Cadaveric	120	High
Rathnakar et al. [67]	2019	Asia	Cadaveric	50	High
Sarvaiya et al. [69]	2019	Asia	Cadaveric	326	High
Victor et al. [86]	2019	Asia	Cadaveric	80	High
Dantas de Lucena et al. [14]	2020	South America	Cadaveric	49	High
Disanayake et al. [15]	2020	Asia	Cadaveric	29	Low
Knezi et al. [34]	2020	Europe	Cadaveric	50	Low
Priya and Jain [63]	2020	Asia	Cadaveric	70	High
Rafi et al. [64]	2020	Asia	Cadaveric	50	Low
Rafi et al. [64]	2020	Asia	Imaging	53	Low
Agrawal et al. [4]	2021	Asia	Cadaveric	180	High
Gautam [22]	2021	Asia	Cadaveric	24	High
Karunakaran and Mohanraj [32]	2021	Asia	Cadaveric	20	High
Muche [49]	2021	Africa	Cadaveric	90	High
Thunyacharoen and Mahakkanukrauh [81]	2021	Asia	Cadaveric	120	Low
Uabundit et al. [82]	2021	Asia	Cadaveric	124	High
Gupta et al. [24]	2022	Asia	Cadaveric	40	High
Li et al. [37]	2022	Asia	Cadaveric	250	High
Prasad and Rout [62]	2022	Asia	Cadaveric	64	High
Saygin et al. [73]	2022	Asia	Cadaveric	107	High
Yavad et al. [90]	2022	Asia	Cadaveric	68	High
Aggarwal et al. [3]	2023	Asia	Cadaveric	56	High
Jan [30]	2023	Asia	Cadaveric	30	High
Nabukalu et al. [52]	2023	Africa	Cadaveric	65	High
Ozor et al. [58]	2023	Africa	Cadaveric	50	High
Roy et al. [68]	2023	Asia	Cadaveric	240	High
Singh et al. [78]	2023	Asia	Cadaveric	115	High
Mahlalela et al. [43]	2024	Africa	Cadaveric	36	Low
Sharma et al. [74]	2024	Asia	Cadaveric	40	High

The most common pattern of the PT morphology was the sphenoparietal type, with a pooled prevalence of 78.54% (95%CI: 76.56–80.46%). The epipteric type was the second most common morphological pattern, with a pooled prevalence of 8.41% (95%CI: 6.70-10.29%). The frontotemporal type was estimated at 5.74% (95%CI: 4.53–7.08%), and the stellate type at 4.26% (95%CI: 3.19–5.47%) (Table 2). A significant difference was observed in the geographical distribution of epipteric, frontotemporal, and stellate PT morphology (Table 3). The results and subgroup analysis of each type according to nationality, sex, side, type of study, and sample size are summarized in Tables 2 and 3, and 4. Between studies, the symmetrical PT morphology (the same type bilaterally) was identified with a pooled prevalence of 82.82% (95%CI: 76.28–88.52), and the asymmetrical PT morphology (different type bilaterally) was estimated at 16.56% (95%CI: 10.93–23.05%). Also, it is essential to highlight that the DOI plot with the LFK index did not indicate a small study effect for the estimated pooled prevalence (Supplementary Material).

**Table 2:** The pterion morphological types (number of studies = 79, number of skulls’ sides = 18694 for all variables). Detailed overview with Higgins I^2^ statistics for heterogeneity quantification and Cochran’s Q test (p-value) for heterogeneity

Pterion Types	Pooled Prevalence (%)	95% CI	I^2^ (%)	*p*-value
Sphenoparietal Pterion	78.54%	76.56–80.46%	90.0%	< 0.0001
Epipteric Pterion	8.41%	6.70-10.29%	94.6%	< 0.0001
Frontotemporal Pterion	5.74%	4.53–7.08%	92.1%	< 0.0001
Stellate Pterion	4.26%	3.19–5.47%	92.4%	< 0.0001

**Table 3:** The pterion morphology types, according to subgroup analysis. All the values are presented in pooled prevalence

Pooled Prevalence (%)	Sphenoparietal Pterion	Epipteric Pterion	Frontotemporal Pterion	Stellate Pterion	
Europe (k = 2)	73.6%	14.60%	0.50%	7.78%	
Africa (k = 10)	76.28%	5.79%	12.25%	4.29%	
Asia (k = 64)	78.97%	8.49%	5.18%	4.37%	
America (k = 1)	85.71%	8.16%	3.06%	3.06%	
Australia (k = 2)	76.18%	17.62%	7.26%	0.42%	
p-value	0.3367	< 0.0001*	< 0.0001*	< 0.0001*	
Anatomical Study (k = 77)	78.31%	8.40%	5.84%	4.39%	
Imaging Study (k = 2)	87.29%	8.80%	2.61%	0.62%	
p-value	0.1703	0.9179	0.2145	0.0279*	
Sample size over 100 (k = 48)	77.77%	9.70%	5.04%	4.43%	
Sample size under 100 (k = 31)	79.88%	6.38%	7.15%	3.93%	
p-value	0.4198	0.1046	0.0752	0.8880	
Left	78.13%	7.31%	5.57%	3.88%	
Right	79.27%	7.06%	4.85%	4.19%	
p-value	0.5374	0.8527	0.4980	0.7600	
Male	76.53%	7.50%	4.77%	3.71%	
Female	71.52%	8.81%	8.65%	3.40%	
p-value	0.3643	0.5979	0.0593	0.9388	

**Table 4:** The pterion morphometric measurements. Detailed overview with Higgins I^2^ statistics for heterogeneity quantification and Cochran’s Q test (p-value) for heterogeneity. MPZ- midpoint of zygomatic arch, FZMS- frontozygomatic suture, MP- mastoid process, EAM- external acoustic meatus

Distance	Pooled Mean (mm)	95% CI	I^2^ (%)	*p*-value
Pterion-MPZ Total	38.66 mm	37.28–40.04 mm	98.5%	< 0.0001
Pterion-MPZ Left	37.97 mm	36.67–39.28 mm	99.3%	< 0.0001
Pterion-MPZ Right	38.48 mm	37.17–39.78 mm	99.3%	< 0.0001
Pterion-FZMS Total	31.31 mm	29.89–32.71 mm	98.8%	< 0.0001
Pterion-FZMS Left	31.43 mm	30.26–32.59 mm	99.7%	< 0.0001
Pterion-FZMS Right	31.83 mm	30.70–32.97 mm	99.6%	< 0.0001
Pterion-MP Total	81.70 mm	78.62–84.78 mm	98.0%	< 0.0001
Pterion-MP Left	79.86 mm	78.63–81.08 mm	98.1%	< 0.0001
Pterion-MP Right	80.32 mm	78.94–81.70 mm	98.2%	< 0.0001
Pterion-EAM Total	53.30 mm	50.95–55.65 mm	94.6%	< 0.0001
Pterion-EAM Left	49.23 mm	42.26–56.21 mm	99.8%	< 0.0001
Pterion-EAM Right	53.36 mm	51.90–54.83 mm	99.3%	< 0.0001

**Fig. 3:**
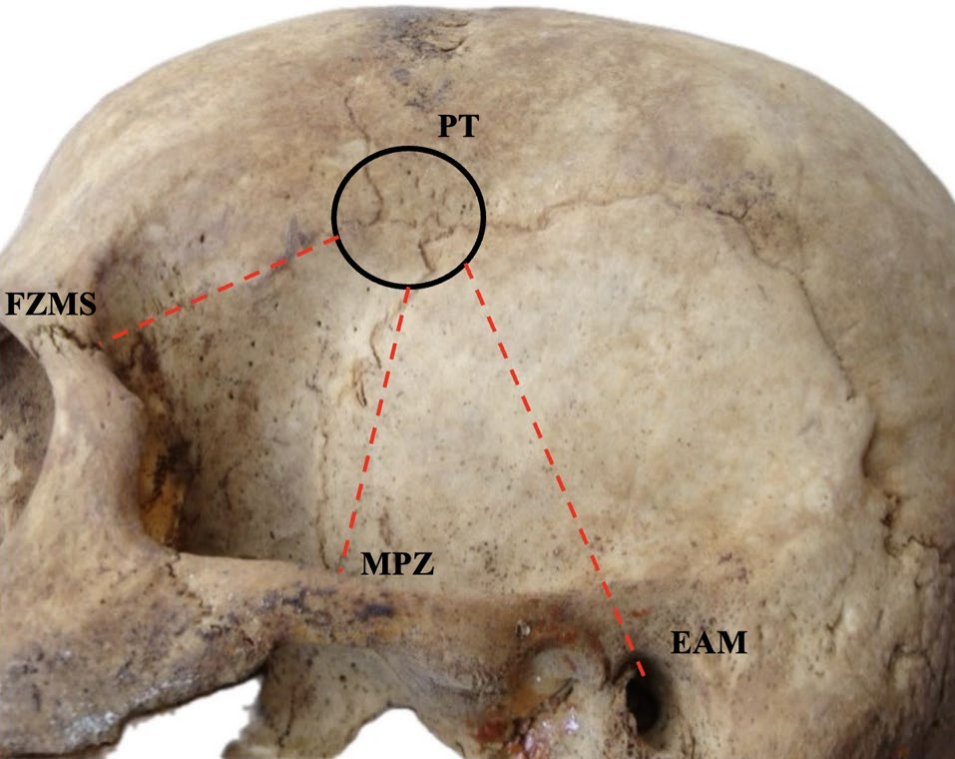
The morphometric measurements from the pterion (PT). MPZ- midpoint of zygomatic arch, FZMS- frontozygomatic suture, EAM- external acoustic meatus

Regarding PT morphometry (Fig. 3), the results of the pooled mean distances per side, are summarized in Table 4. The distances PT-MPZ was estimated with pooled mean of 38.66 mm (95%CI: 37.28–40.04 mm), the PT-MP was calculated at 81.70 mm (95%CI: 78.62–84.78 mm), the PT-FZMS was estimated at 31.31 mm (95%CI: 29.90–32.71 mm) and the PT-EAM was calculated with a pooled prevalence of 53.30 mm (95%CI: 50.95–55.65 mm).

## Discussion

In the current systematic review with meta-analysis, we aimed to evaluate the PT morphology and its distance from noteworthy landmarks. Murphy [h^50^] first described four different patterns of PT morphology. The sphenoparietal, frontotemporal, stellate, and epipteric types of PT were the four patterns proposed by Murphy [50] that were followed by most of the published studies. Herein, we established their pooled prevalence. Hence, the sphenoparietal type corresponds to the PT typical anatomy (78.54% pooled prevalence), while the epipteric (8.41%), the frontotemporal (5.74%), and the stellate (4.26%) types are PT variants. Moreover, we observed a significant difference between the PT morphology and the nationality, which was also raised by Natsis et al. [55] study; however, the subgroup analysis based on the geographical distribution did not reach three articles per continent, which is the minimum threshold according to Fu et al. [20]. The symmetrical appearance of PT morphology (same type bilateral) was estimated at 82.82%, while an asymmetry was identified at 16.56%. Recently, Uabundit et al. [82] proposed a fifth morphological pattern for the PT, based on their findings, the “synostotic” PT presented with complete synostosis of the PT sutures. This type was identified in 18.5% of their sample but was not reported by other studies and hence was excluded from the present meta-analysis. Nevertheless, PT distance from other landmarks is clinically essential for adequate localization. Herein, we presented the pooled mean distance of PT from the MPZ, the FZMS, the MP, and the EAM. Using a machine learning approach, Uabundit et al. [82] correlated the PT distances with the sex and age of skulls. The accuracy of sex prediction was estimated at approximately 80%, while the age prediction did not show encouraging results [82]. Therefore, the PT distances from various landmarks could be affected by sex; however, only a few studies investigated this relationship [55, 82], and it was excluded from the meta-analysis. Ma et al. [41] reported the variability of the PT description in classical anatomical and surgical books, mainly for the PT distance from other landmarks.

The classification of the epipteric pterion type is based on the location and the number of EBs. Only a few studies have previously indicated the presence of this morphological type; and hence, it could not be included in the present meta-analysis. Natsis et al. [55] proposed a novel method to anatomically define the EBs based on their number and the name of the sutures articulating them. A single EB articulating with four sutures was described as a “typical quadrilateral.” A single bone articulating with three sutures was subclassified as superior, inferior, anterior, and posterior based on its location, while a bone articulating with two sutures was defined as “bisutural” [55]. A fusion failure between the posterosuperior border of the greater sphenoidal wing and the rest of the greater wing could result in the appearance of an EB around the 4th month of development [65].

The PT is an important landmark in neurosurgery for various procedures. However, this skull area is relatively thin and fragile. It is located above the anterior branch of the middle meningeal artery, the lateral sulcus of the brain, and Broca’s motor speech area [3]. The importance of the identification of PT prior to craniotomy is to avoid opening the lateral wall of the orbit (rare and not very serious) and to anticipate the risk of injury to the middle meningeal artery (frequent but with exceptional consequences). The most common cause of acute traumatic epidural hematoma is the anterior branch of the middle meningeal artery. Therefore, understanding the typical and variant anatomy of the PT is crucial for neurosurgeons during hematoma evacuation [41, 76]. The 10 mm radius circle with the midpoint of the PT was observed to overlap the anterior branch of a middle meningeal artery in 68% of cases [41]. Interestingly, during a large retrospective magnetic resonance angiography study, it was reported that the ophthalmic artery can be seen from the middle meningeal artery, with a prevalence of 1.45% [83]. In cases where this variation exists, a pterional craniotomy could cause occlusion of the ophthalmic artery, leading to blindness [75].

A tailored pterional approach is used for various neurosurgical procedures, including the treatment of aneurysms, sellar and frontotemporal lesions, suprasellar lesions, and sphenoid ridge meningiomas [41]. This approach is preferred for anterior and middle skull base surgery, most anterior circulation aneurysms, and selected median and paramedian, including the upper part of the posterior circulation [38–40]. After making the skin incision and visualizing the skull, the sutures can serve as landmarks, with the PT being the joining point of several sutures [38–40]. The craniotomy is typically performed 5 mm behind the connection of the frontozygomatic, sphenozygomatic, and fronto-sphenoidal sutures [38–40]. It’s important to note that EBs can be confusing for neurosurgeons during surgery, leading them to mistakenly diagnose the pterion as the most anterior junction of the sutures [18]. The pterional craniotomy should be tailored to the specific underlying pathology, making it essential to consider the morphological variability of the pterion. For smaller, unruptured paraclinoid aneurysms and unruptured middle cerebral artery bifurcation aneurysms, a smaller craniotomy is preferable. In contrast, larger craniotomies are necessary for ruptured aneurysms due to the presence of brain edema. This tailored approach ensures optimal surgical access while minimizing risks associated with the procedure [38–40]. Amini et al. [7] suggested that the lateral supraorbital approach for middle cerebral aneurysms offers several benefits, including reduced operation time and fewer complications compared to the personalized approach.

The present study has some limitations. Firstly, there was considerable heterogeneity and a high risk of bias, which is common in anatomical reviews [26, 27]. Unfortunately, there were not enough articles on EB classification to be included in the meta-analysis. Additionally, the subgroup analysis based on nationality and study type did not meet the minimum requirement of three articles per parameter, as recommended by Fu et al. [20]. These factors may affect the generalizability and precision of our findings. Most of the studies were performed on Asian population, further research on other populations will increase the current knowledge.

## Conclusions

The predominant pterion type observed was the sphenoparietal, with an overall prevalence of 78.54%. The epipteric, frontotemporal, and stellate types were identified with frequencies of 8.41%, 5.74%, and 4.26%, respectively. This study provides essential anatomical insights by accurately mapping the pterion’s location in relation to these cranial landmarks. These findings are crucial for neurosurgical planning, particularly for procedures involving the anterior and middle cranial fossae. The detailed anatomical data enhance the precision and safety of neurosurgical procedures, ultimately contributing to improved patient outcomes.

## Electronic supplementary material

Below is the link to the electronic supplementary material.

Supplementary Material 1

## Data Availability

No datasets were generated or analysed during the current study.
