# Supplementary material for: Unveiling pterion variability: a meta-analytic approach to enhance neurosurgical precision
Source: Surg Radiol Anat. 2025 Jan 28;47(1):66. doi: 10.1007/s00276-025-03571-6 (PMC11775020; doi:10.1007/s00276-025-03571-6)
Supplement: Supplementary file 1 — Supplementary Material 1 [file 276_2025_3571_MOESM1_ESM.docx]

**Supplementary Material**

Figures from the statistical meta-analysis using the R programming software.


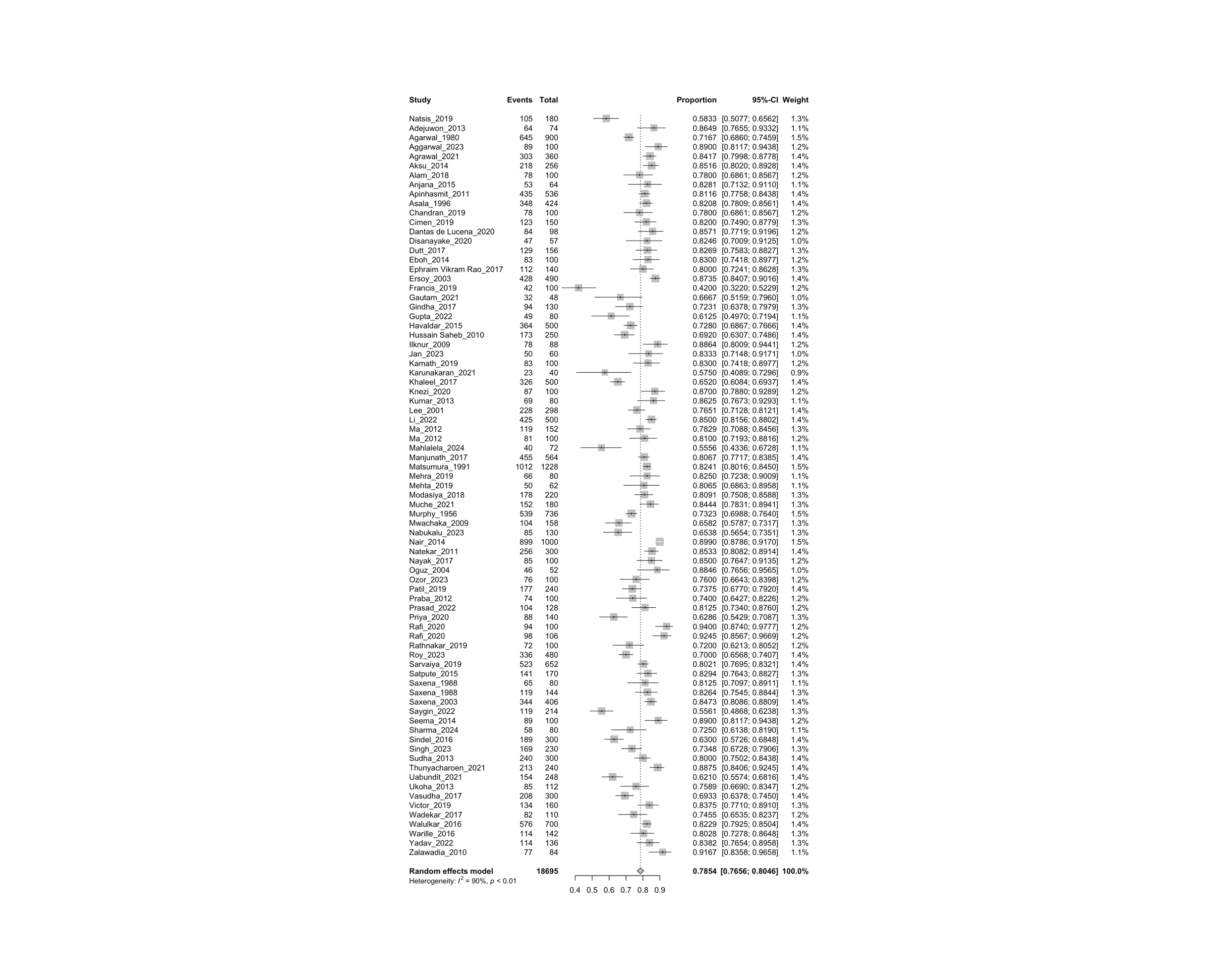


**Figure 1.** Forest plot of the sphenoparietal pterion morphology pooled prevalence.


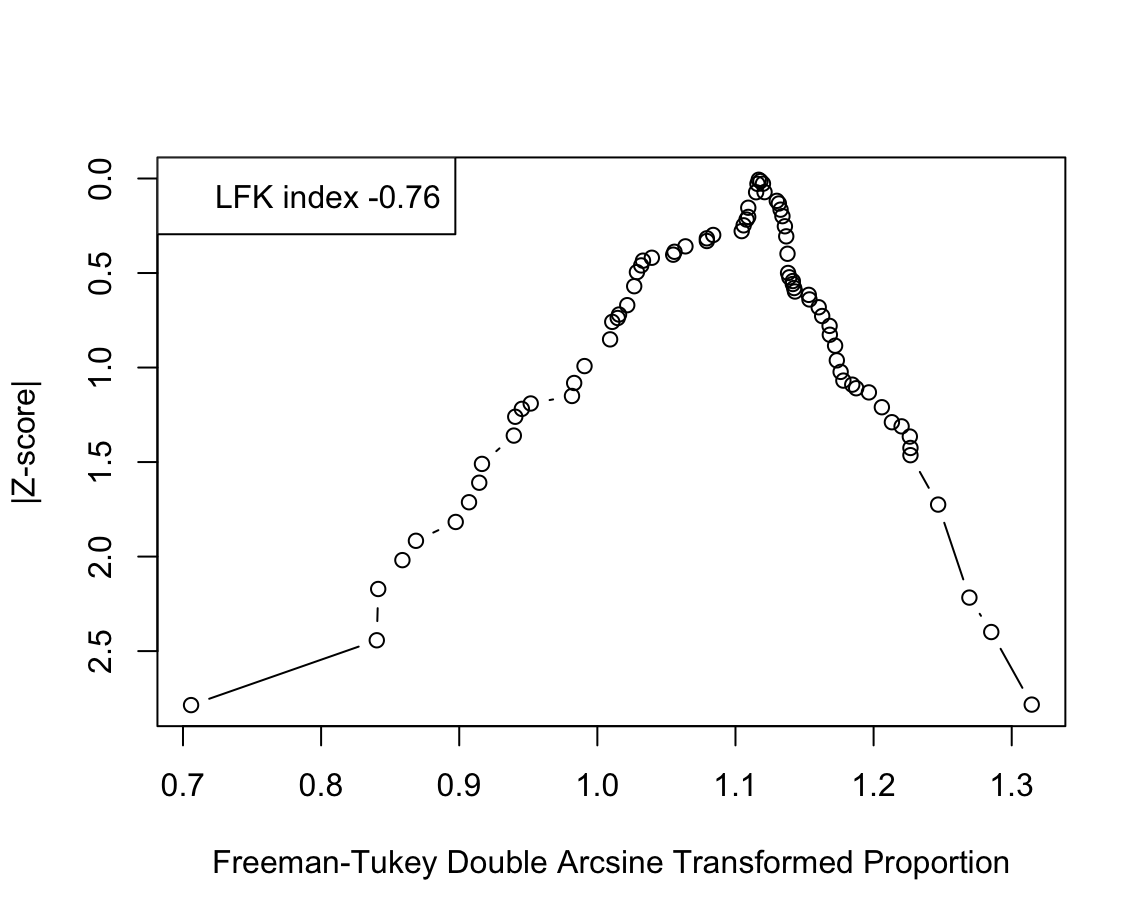


**Figure 2.** DOI plot with LFK index for sphenoparietal pterion pooled prevalence possible small study effect.


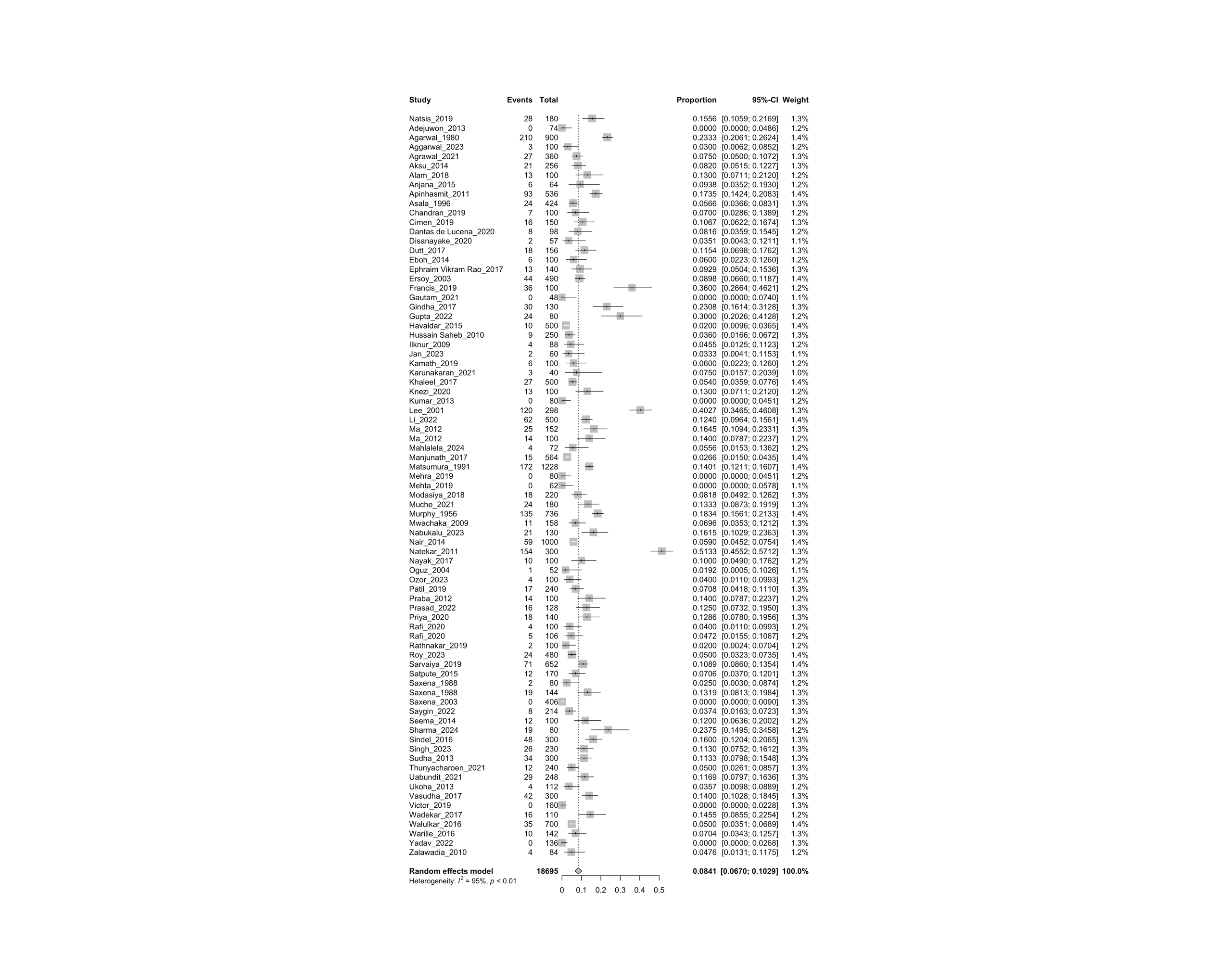


**Figure 3.** Forest plot for epipteric pterion morphology pooled prevalence


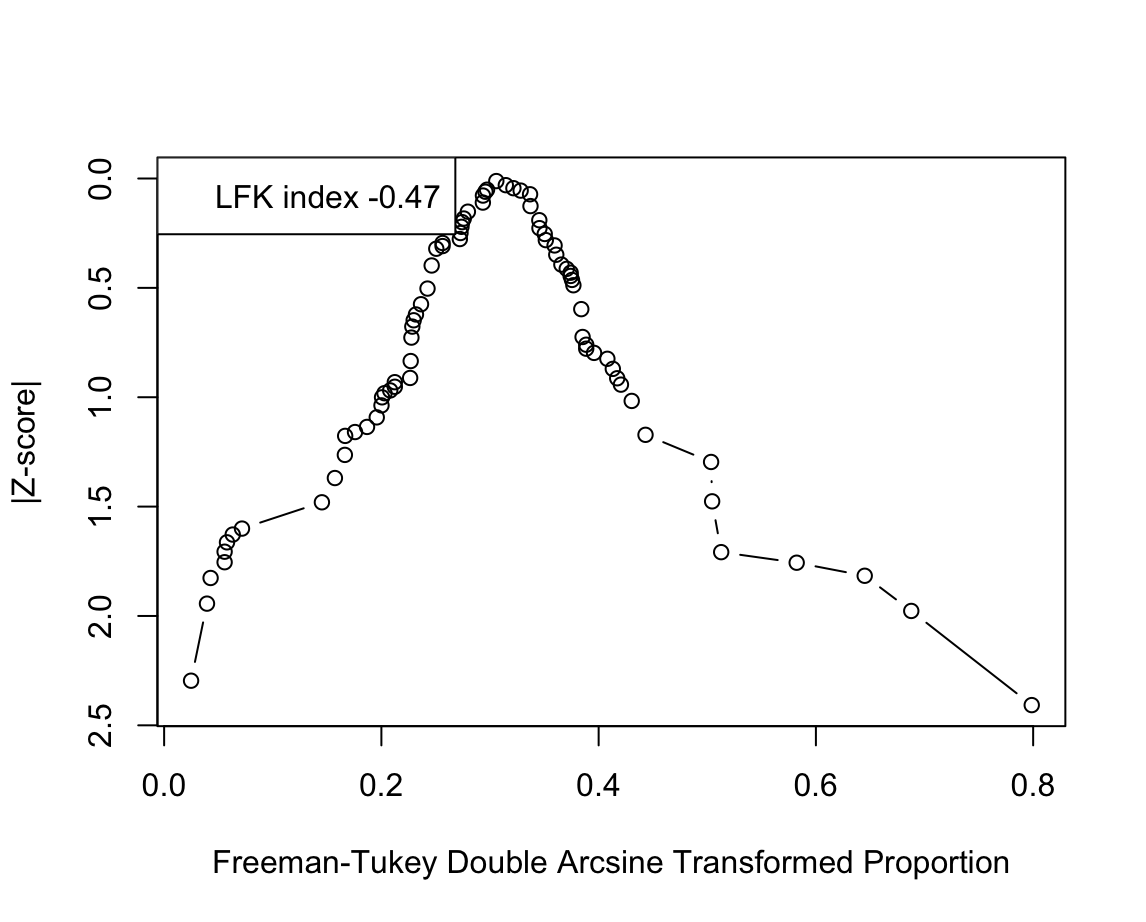


**Figure 4**. DOI plot with LFK index for epipteric pterion pooled prevalence possible small study effect.


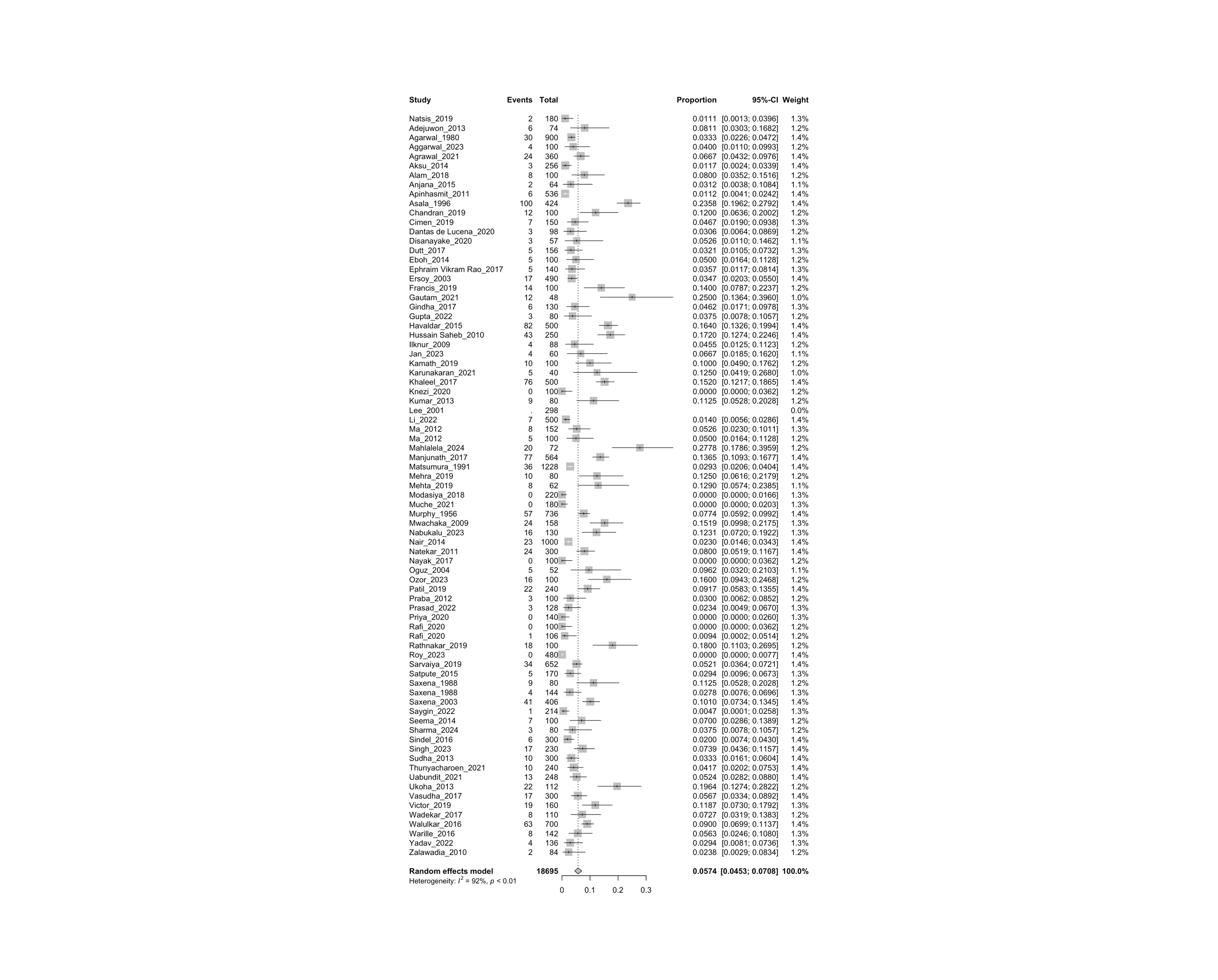


**Figure 5.** Forest plot for frontotemporal pterion morphology pooled prevalence.


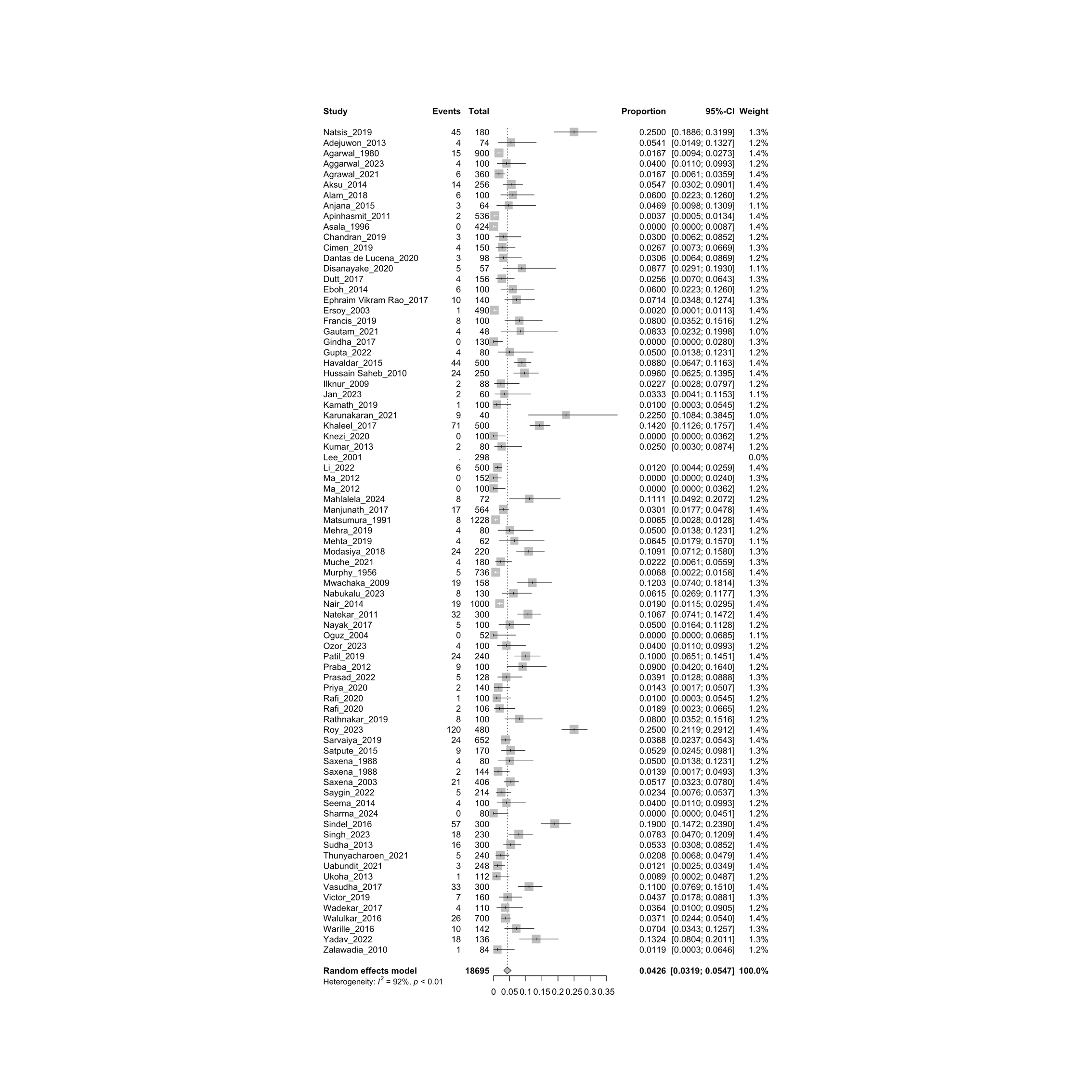


**Figure 6.** Forest plot for stellate pterion morphology pooled prevalence.
